# Salivary Biomarkers for Oral Squamous Cell Carcinoma Diagnosis and Follow-Up: Current Status and Perspectives

**DOI:** 10.3389/fphys.2019.01476

**Published:** 2019-12-10

**Authors:** Marta Cristaldi, Rodolfo Mauceri, Olga Di Fede, Giovanna Giuliana, Giuseppina Campisi, Vera Panzarella

**Affiliations:** ^1^Department of Surgical, Oncological and Oral Sciences, University of Palermo, Palermo, Italy; ^2^Department of Biomedical and Dental Sciences and Morphofunctional Imaging, University of Messina, Messina, Italy

**Keywords:** liquid biopsy, salivary biomarkers, circulating tumor DNA, extracellular vesicles, microRNAs, early diagnosis, prognosis, oral squamous cell carcinoma

## Abstract

Oral cancer is the sixth most common cancer type in the world, and 90% of it is represented by oral squamous cell carcinoma (OSCC). Despite progress in preventive and therapeutic strategies, delay in OSCC diagnosis remains one of the major causes of high morbidity and mortality; indeed the majority of OSCC has been lately identified in the advanced clinical stage (i.e., III or IV). Moreover, after primary treatment, recurrences and/or metastases are found in more than half of the patients (80% of cases within the first 2 years) and the 5-year survival rate is still lower than 50%, resulting in a serious issue for public health. Currently, histological investigation represents the “gold standard” of OSCC diagnosis; however, recent studies have evaluated the potential use of non-invasive methods, such as “liquid biopsy,” for the detection of diagnostic and prognostic biomarkers in body fluids of oral cancer patients. Saliva is a biofluid containing factors such as cytokines, DNA and RNA molecules, circulating and tissue-derived cells, and extracellular vesicles (EVs) that may be used as biomarkers; their analysis may give us useful information to do early diagnosis of OSCC and improve the prognosis. Therefore, the aim of this review is reporting the most recent data on saliva biomarker detection in saliva liquid biopsy from oral cancer patients, with particular attention to circulating tumor DNA (ctDNA), EVs, and microRNAs (miRNAs). Our results highlight that saliva liquid biopsy has several promising clinical uses in OSCC management; it is painless, accessible, and low cost and represents a very helpful source of diagnostic and prognostic biomarker detection. Even if standardized protocols for isolation, characterization, and evaluation are needed, recent data suggest that saliva may be successfully included in future clinical diagnostic processes, with a considerable impact on early treatment strategies and a favorable outcome.

## Introduction

Oral cancer is one of the most common cancers in the world, representing a serious problem for global health (Shah and Gil, 2009). Roughly 90% of oral cancers histologically originate from squamous cells and are classified as oral squamous cell carcinoma (OSCC) (Rivera, 2015; Mascitti et al., 2018).

Despite current advancements in cancer prevention strategies and therapeutic approaches, the prognosis of OSCC-affected patients is still poor; this mainly depends on late diagnosis in an advanced stage (i.e., III or IV) (Rivera, 2015; Mascitti et al., 2018). Moreover, after primary treatment, recurrences and/or metastases are found in more than half of the patients (80% of cases within the first 2 years), and the 5-year survival rate is still lower than 50%, resulting in a serious issue for public health; as a result, in the latest decades, the annual mortality rate has remained unchanged at 145,000 cases (Grobe et al., 2013; Panzarella et al., 2014; Rivera, 2015).

To date, the “gold standard” for OSCC diagnosis is represented by a clinical oral examination integrated by a histological investigation on tissue biopsy of suspicious lesions (Fuller et al., 2015). However, cancer research is currently focusing on finding less invasive and cost-effective methods to provide a more comprehensive view of the cancer profile, also to more easily monitor its evolution and therapeutic response (Siravegna et al., 2017; Wang J. et al., 2017). In this regard, many non-invasive techniques such as liquid biopsy have been recently proposed as supportive tools for diagnosis, prognosis, and follow-up of OSCC (Dionne et al., 2015; Fuller et al., 2015; Aro et al., 2017).

As is widely known, cancers developed from different types of DNA alterations which are classified based on their effects on structure: small-scale genetic alterations, which comprise different types of point mutations (e.g., DNA base insertions, substitutions, and deletions), and large-scale gene alterations such as DNA rearrangements (e.g., gene duplications/deletions, chromosomal translocations/inversions, and loss of heterozygosity) (Stadler et al., 2010). It is noteworthy that the changes in DNA genetic and epigenetic structure (i.e., altered function of factors implicated in gene expression) can be detected by several biomarkers as circulating tumor cells (CTCs), circulating tumor DNA (ctDNA), microRNAs (miRNAs), and extracellular vesicles (EVs), which can be found in several biological fluids such as blood, serum, plasma, pleural fluid, urine, and saliva (Kaczor-Urbanowicz et al., 2017). The evaluation of these cancer biomarkers in liquid biopsy has the advantage of providing a real-time picture of primary and metastatic tumors at different time points, giving information about tumor and tumor burden and early evidence of drug resistance and tumor recurrence (Di Meo et al., 2017). Moreover, liquid biopsy analysis allows defining of the DNA molecular profile of cancer patients, whose inclusion in the tumor, node, and metastasis staging system may help to develop more highly personalized therapies and decrease risks of inappropriate treatments (Xu et al., 2017).

Oral squamous cell carcinoma is strictly associated with the oral microenvironment, resulting in a direct contact with saliva, an acidic biological fluid derived from secretion of major and minor salivary glands, and involved in many physiological and pathological processes (Kaufman and Lamster, 2002; Nieuw Amerongen and Veerman, 2002; Chiappin et al., 2007; Jankowska et al., 2007; Lee and Wong, 2009). In recent decades, saliva has been widely investigated as a promising source of OSCC biomarkers for liquid biopsy (Kaczor-Urbanowicz et al., 2017). Employing saliva in liquid biopsy for OSCC management has many advantages over other specimens: (i) it is considered “the mirror of the body,” reflecting any physiological and pathological change on local and distant sites of the body; (ii) it represents an easier, quicker, and more accessible screening tool (Kaczor-Urbanowicz et al., 2017); and (iii) it gives the opportunity to collect larger volumes of samples for examination, to easily repeat analysis, and to monitor OSCC over time (Kaczor-Urbanowicz et al., 2017; Gai et al., 2018; Khurshid et al., 2018).

In light of the above, this narrative review aims to describe the recent data regarding the employment of different salivary biomarkers in liquid biopsy for OSCC diagnosis, prognosis, follow-up, and patient response to therapy. Moreover, we discuss the current challenges and future perspectives concerning the use of salivary biomarkers in OSCC management.

## Salivary Biomarkers and OSCC

### Circulating Tumor DNA

During physiological cellular turnover or in particular pathological conditions, apoptotic and necrotic cells release debris and DNA/RNA molecules into body fluids. In physiologic conditions, cell-derived molecules and debris are removed by phagocytes; instead, in cancer patients, this mechanism results in impairment, inducing the accumulation of cell-free DNA (cfDNA) in the tissue microenvironment and biological fluids (Jahr et al., 2001; Chan et al., 2004; Mouliere et al., 2011). As a result, cancer patients have increased levels of cfDNA in body fluids (Diehl et al., 2008; Kohler et al., 2011). cfDNA released from cancer cells, also known as ctDNA, can be distinguished from cfDNA physiologically released from non-cancer cells through several features: biofluid concentration, somatic mutations, and overall size. Because of random partial digestion of genomic DNA, cfDNA from apoptotic cells is measured at 180–200 base pairs; necrosis or autophagy during the cancer process usually generates larger DNA molecules, from 100 to 400 base pairs (Wang et al., 2003; Diehl et al., 2008; Kohler et al., 2011).

It is noteworthy that many studies also showed that ctDNA in cancer patients reflects the genetic and epigenetic alterations found in tissue samples of malignant lesions (Ignatiadis et al., 2015; Cheng et al., 2016; Qin et al., 2016); in addition, many other cancer characteristics such as size, cellular turnover, stage, vascularity, and drug response are associated with ctDNA concentrations (Chang et al., 2017).

Several mechanisms have been suggested to explain the release of ctDNA in body fluids of cancer patients, even if they remain dubious. It could be the result of accelerated metabolism of cancer cells, responsible for the increased number of apoptotic/necrotic cells (Shukla et al., 2013; Francis and Stein, 2015; Volik et al., 2016; Payne et al., 2018). Cancer cells released from primary tumor mass and disseminating throughout body fluids (e.g., CTCs) could actively release fragments of cellular nucleic acids, which, when internalized from non-cancer cells, induce neoplastic transformation (Ilie et al., 2014; Cheng et al., 2016). Cancer transformation can be also the result of the internalization of vesicles (e.g., EVs), containing ctDNA and actively released from cancer cells, from healthy cells (Lau et al., 2013; Kaczor-Urbanowicz et al., 2017).

ctDNA is mainly released into the bloodstream; however, it can also be found in other body fluids. For instance, by means of salivary gland ultrafiltration, passive diffusion, or active transport, ctDNA can easily reach saliva from a local site and bloodstream, carrying information regarding primary tumors and/or metastasis (Pu et al., 2016; Kaczor-Urbanowicz et al., 2017; Wang X. et al., 2017). Thanks to less dilution and contamination, the analysis of the ctDNA in saliva is much more sensitive than that in the bloodstream (Peng et al., 2017).

As described in Table 1, many studies have applied salivary ctDNA in the management of head and neck squamous cell carcinoma (HNSCC) (Liao et al., 2000; El-Naggar et al., 2001; Viet and Schmidt, 2008; Sethi et al., 2009; Demokan et al., 2010; Carvalho et al., 2011; Guerrero-Preston et al., 2011; Sun et al., 2012; Rettori et al., 2013; Ramadoss et al., 2015; Wang et al., 2015; Ferlazzo et al., 2017; Lacombe et al., 2017). When HNSCC patients with tumors at the oral cavity, oropharynx, larynx, and hypopharynx were enrolled at the early stages (I and II) and late stages (III and IV), ctDNA was detected, respectively, in 100% of HNSCC patients enrolled at the early stages and in 95% enrolled at the late stages, with an enrichment in saliva of all patients affected by OSCC; this result is very specific for OSCC detection. Moreover, being postsurgically found in 100% of patients who subsequently had a clinical recurrence and in any patients without recurrence, it could be also a valuable biomarker for oral cancer follow-up and surveillance (Wang et al., 2015). Interestingly, when the OSCC-leading genetic alterations in ctDNA of saliva and tissue samples were analyzed, a correlation in loss of heterozygosity and p53 mutations was found, demonstrating that saliva can be a reliable and non-invasive alternative to tissue biopsy for OSCC detection (Liao et al., 2000; El-Naggar et al., 2001; Ramadoss et al., 2015).

**TABLE 1:** Diagnostic, prognostic, and follow-up applications of ctDNA in OSCC, based on population study.

**Authors (year)**	**Title**	**Cases**	**Controls**	**Type of alteration**	**Application**	**Results**
Liao et al., 2000	Mutation of p53 gene codon 63 in saliva as a molecular marker for oral squamous cell carcinomas	10 OSCC	27	DNA point mutation	Diagnosis	Of the OSCCs, 62.5% harbor C-deletion in exon 4 codon 63 from preoperative saliva samples. TSG p53 exon 4 codon 63 mutation is a good biomarker for OSCC diagnosis.
El-Naggar et al., 2001	Genetic heterogeneity in saliva from patients with oral squamous carcinomas: implications in molecular diagnosis and screening	40 OSCC	10	DNA microsatellite	Diagnosis	A correlation on DNA microsatellite alterations between saliva and tumor specimens was found. The saliva ctDNA can be used as a genetic biomarker for early detection of OSCC.
Viet and Schmidt, 2008	Methylation array analysis of preoperative and postoperative saliva DNA in oral cancer patients	13 OSCC	10	DNA hypermethylation	Diagnosis and prognosis	Sites highly methylated in the tissue and preoperative saliva samples, but not methylated in the postoperative saliva samples or in normal subjects, were found. A genetic classifier consisting of specifically methylated gene loci was generated and can be used as a composite biomarker for early diagnosis and prognosis of OSCC.
Sethi et al., 2009	Non-invasive molecular detection of head and neck squamous cell carcinoma: an exploratory analysis	27 HNSCC (7 OSCC)	10	CNV (loss and gain)	Diagnosis	Gain of PMAIP1 (18q21.31) solely or in conjunction with gain of PTPN1 (20q13.13), detected in saliva DNA, differentiated 100% of HNSCC cases from normal controls with high sensitivity and specificity, with no tumor site differentiation. However, it has clinical utility for non-invasive HNSCC diagnosis and screening, including OSCC.
Demokan et al., 2010	KIF1A and EDNRB are differentially methylated in primary HNSCC and salivary rinses	101 HNSCC (39 OSCC)	76	DNA hypermethylation	Diagnosis	Promoter hypermethylation of K1F1A and EDNRB genes, detected in saliva DNA, is preferentially methylated in salivary rinses from HNSCC compared with healthy patients. In addition, patients with OSCC had higher methylation on the K1F1A promoter. KIF1A and EDNRB are potential biomarkers for HNSCC detection, with K1F1A being more specific for OSCC.
Carvalho et al., 2011	Detection of promoter hypermethylation in salivary rinses as a biomarker for head and neck squamous cell carcinoma surveillance	61 HNSCC (30 OSCC)	–	DNA hypermethylation	Prognosis and follow-up	Of the HNSCC patients analyzed, 54.1% showed methylation in the promoter of at least one of the studied genes (i.e., DAPK, DCC, MINT-31, TIMP3, p16, MGMT, and CCNA1) in saliva ctDNA, with a significantly lower local disease control and overall survival in patients with recurrence. In addition, among all sites analyzed, a higher percentage of patients with OSCC, with respect to the other sites, showed DNA hypermethylation. Detection of DNA hypermethylation in saliva ctDNA can potentially predict local recurrence and overall survival in OSCC patients.
Guerrero-Preston et al., 2011	NID2 and HOXA9 promoter hypermethylation as biomarkers for prevention and early detection in oral cavity squamous cell carcinoma tissues and saliva	16 OSCC	19	DNA hypermethylation	Diagnosis	The promoter hypermethylation of HOXA9 and NID2 genes detected in tissue, saliva, and serum ctDNA of OSCC patients has a moderate to significant agreement with clinical diagnosis, distinguishing healthy from OSCC patients at the moment of pretreatment. They are potentially useful for OC early detection and prevention.
Sun et al., 2012	Detection of TIMP3 promoter hypermethylation in salivary rinse as an independent predictor of local recurrence-free survival in head and neck cancer	197 HNSCC (53 OSCC)	–	DNA hypermethylation	Prognosis	Promoter hypermethylation of TIMP3 detected in pretreatment salivary rinse ctDNA of HNSCC patients is associated with disease-free survival; even if without tumor site differentiation, hypermethylation of TIMP3 can be considered a salivary independent prognostic factor of HNSCC recurrence, including OSCC.
Rettori et al., 2013	Prognostic significance of TIMP3 hypermethylation in post-treatment salivary rinse from head and neck squamous cell carcinoma patients	146 HNSCC (70 OSCC)	60	DNA hypermethylation	Diagnosis, prognosis, and follow-up	DNA hypermethylation in CCNA1, DAPK, DCC, MGMT, and TIMP3 is strictly correlated to the presence of OSCC tumor. Moreover, 100% of HNSCC patients with TIMP3 DNA hypermethylation, 6 months after treatment, had lower local recurrence-free survival. DNA hypermethylation on CCNA1, DAPK, DCC, MGMT, and TIMP3 genes is a specific OSCC diagnostic biomarker; TIMP3 DNA hypermethylation is an independent prognostic factor to recognize HNSCC patient subgroups with high risk of local recurrence.
Ramadoss et al., 2015	C-deletion in exon 4 codon 63 of p53 gene as a molecular marker for oral squamous cell carcinoma: a preliminary study	20 OSCC	5	DNA point mutation	Diagnosis	PCR data showed the presence of C-deletion in exon 4 of p53 gene in saliva of 100% OSCC. Mutation in exon 4 codon 63 of the p53 gene, evaluated by PCR, is a fast, reliable, accurate, and sensitive molecular method that may be employed as a potential molecular diagnostic marker for OSCC.
Wang et al., 2015	Detection of somatic mutations and HPV in the saliva and plasma of patients with head and neck squamous cell carcinomas	93 HNSCC (3 OSCC)	–	DNA somatic mutations and HPV genes	Diagnosis, prognosis, and follow-up	After evaluation of ctDNA somatic mutations or HPV genes from saliva and plasma of HNSCC patients, saliva ctDNA mutations resulted in 100% specificity for OSCC cancer detection. Moreover, any mutation was identified after surgery in the patients whose tumors did not recur, being considered a valuable diagnostic, prognostic, and follow-up biomarker.
Ferlazzo et al., 2017	Influence of MTHFR genetic background on p16 and MGMT methylation in oral squamous cell cancer	58 OSCC	90	DNA hypermethylation	Diagnosis	There is a higher frequency of p16 and MGMT promoter methylation in OSCC patients than in normal controls. The assessment of DNA methylation rate could represent a powerful diagnostic approach for early detection of cancer.
Lacombe et al., 2017	Analysis of saliva gene expression during head and neck cancer radiotherapy: a pilot study	8 HNSCC (1 OSCC)	–	Aberrant expression	Follow-up	This pilot study shows that salivary gene biomarkers can be detected and their expression modified during head and neck radiotherapy. The increasing expression of the genes CCDKN1A and DDB2, during radiation treatment, is correlated to the cumulative received dose. Saliva is a minimally invasive means of biomarker collection to directly measure radiation dose escalation during treatment.

*OSCC, oral squamous cell carcinoma; ctDNA, circulating tumor DNA; TSG, tumor suppressor gene; HNSCC, head and neck squamous cell carcinoma; CNV, copy number variation; miRNA, microRNA; PCR, polymerase chain reaction; HPV, human papillomaviruses; MTHFR, methylenetetrahydrofolate reductase.*

Among the epigenetic alterations frequently found in salivary ctDNA, gene promoter methylation has been extensively investigated in cancer. Numerous studies, comparing the methylation rate of genes involved in the cell cycle, proliferation, and apoptosis, found a difference in salivary samples of HNSCC patients and healthy subjects, demonstrating that salivary ctDNA methylation could be considered a very promising biomarker for HNSCC management and specifically OSCC diagnosis, prognosis, and follow-up (Viet et al., 2007; Viet and Schmidt, 2008; Sethi et al., 2009; Puttipanyalears et al., 2014). Epigenetic alterations such as DNA methylation rate can be easily detected in salivary ctDNA, proving to be a valuable sensitive and specific marker for OSCC diagnosis and prognosis (Demokan et al., 2010; Pattani et al., 2010; Guerrero-Preston et al., 2011; Ferlazzo et al., 2017). Moreover, the evaluation of the methylation status of genes, involved in OSCC development at diagnosis, seems to be useful as a predictive factor to develop a personalized therapy and assess the patient response during surveillance (Viet and Schmidt, 2008; Lacombe et al., 2017).

### Extracellular Vesicles

As is widely known, EVs represent one of the main mechanisms of intercellular communication (Deutsch et al., 2008). The discovery of cell-derived EVs in saliva able to selectively “package” factors such as DNAs, RNAs, miRNAs, and proteins recently attracted the attention of researchers, being promising additional sources of biomarkers (Valadi et al., 2007; Krief et al., 2011; El Andaloussi et al., 2013; Lötvall et al., 2014; Wong, 2015; Yáñez-Mó et al., 2015). To date, the most investigated vesicles in cancer development and progression are exosomes and microvesicles, small membrane vesicles similar in some structural and functional elements but distinguished by means of size and cellular release processes (Wong, 2006; Pfaffe et al., 2011; Corrado et al., 2013; Kastelowitz and Yin, 2014).

Many studies demonstrated that EVs, transferring protein and nucleic acids able to support or inhibit tumorigenesis, are implicated in tumor microenvironments (Kalluri, 2016; Ruivo et al., 2017); additionally, it has been recently shown that EVs may have a role in oral cancers (Gai et al., 2018; Han et al., 2018). Differences in relative size, proteome signature, and expression of exosomal markers have been highlighted between OSCC parental and metastatic cells; moreover, OSCC parental cells release exosomes with oncogenic markers able to influence the surrounding tumor microenvironment. The evaluation of exosomal markers from OSCC patients can support diagnosis, prognosis, and assessment of patient response to therapy, detecting potential tumor recurrence (Byun et al., 2015; Languino et al., 2016; Li et al., 2016; Liu et al., 2017; Ono et al., 2018).

Nevertheless, few studies have evaluated the role of salivary EVs in OSCC management (Table 2; Winck et al., 2015; Zlotogorski-Hurvitz et al., 2016). Salivary tumor-derived exosomes have different morphological and molecular features compared to healthy saliva samples and can detect cancer and malignant transformations early in high-risk patients (Zlotogorski-Hurvitz et al., 2016; Malonek et al., 2018). In the comparison of the proteomic analysis of whole saliva from healthy and OSCC patients, it was also shown that the qualitative and quantitative features of saliva EVs may support OSCC diagnosis and give information regarding the prognosis of cancer patients. The proteome of salivary EVs shows the presence of proteins implicated in cancer inflammatory response, transport of metals, and cellular growth and proliferation, allowing the classification of OSCC patients and the ability to give prognosis information with a high percentage of accuracy (Winck et al., 2015).

**TABLE 2:** Diagnostic and prognostic application of EVs in OSCC, based on population study.

**Authors (year)**	**Title**	**Cases**	**Controls**	**Type of alteration**	**Application**	**Results**
Winck et al., 2015	Insights into immune responses in oral cancer through proteomic analysis of saliva and salivary extracellular vesicles	24 OSCC	10	Aberrant expression	Prognosis	The proteomic data from whole saliva and saliva EVs of OSCC and healthy patients allow us to classify OSCC patients with 90% accuracy, being considered valuable additional parameters/biological biomarkers to define the prognosis in OSCC patients.
Zlotogorski-Hurvitz et al., 2016	Morphological and molecular features of oral fluid-derived exosomes: oral cancer patients versus healthy individuals	36 OC (OSCC not specified)	25	Altered exosome morphology and protein composition	Diagnosis	Saliva from OC patients contains morphologically and molecularly different exosomes with respect to healthy subjects. In particular, the decrease in the expression of CD9 and CD81 rather than the increase of CD63 expression can be an indicator of OC, even in the very early stages of the disease, in high-risk patients without clear clinical sign.

*EV, extracellular vesicle; OC, oral cancer; OSCC, oral squamous cell carcinoma.*

### MicroRNAs

MicroRNAs are small single-strand RNA molecules (19–21 nucleotides), transcribed by cellular polymerase RNA and submitted to a double sequential cut: in the nucleus (primary miRNA) and cytoplasm (precursor miRNA). Then, depending on the complementarity level with the target, they cleave a messenger RNA (mRNA) or inhibit its translation (Bartel, 2004; Perron and Provost, 2008).

MicroRNAs can be released into body fluids as cell-free miRNAs, associated with RNA-binding proteins or selectively packaged into EVs (Larrea et al., 2016). Their involvement in tumor microenvironments has been widely investigated, demonstrating that the expression of miRNAs could be associated with the deregulation of oncosuppressors or oncogenes, contributing to tumor development or inhibition. Moreover, they were unchanged in tissues and body fluids, and their expression is frequently tumor specific; for these reasons, the possibility to analyze miRNA profiles associated with cancer patients represents a new paradigm in biomarker discovery for cancer clinical diagnostics. Specifically, with regard to HNSCC cancers strictly associated with saliva, the evaluation of miRNAs released by OSCC cells in saliva represents a promising perspective for OSCC management (Momen-Heravi et al., 2014).

Over the years, many studies and reviews summarized the updates on evaluation of miRNA expression alteration for diagnosis and risk stratification of oral cancers, finding that many miRNAs are differentially expressed between healthy and OSCC patients (Table 3; Park et al., 2009; Wiklund et al., 2011; Liu et al., 2012; Yang et al., 2013; Momen-Heravi et al., 2014; Al-malkey and Abbas, 2015; Zahran et al., 2015; Duz et al., 2016; Greither et al., 2017). The different salivary expression of miRNAs, as miR-125, miR- 200a, miR-21, miR-145, miR-200a, miR-93, miR-375, and miR-184 in OSCC patients compared to the healthy subjects, has been demonstrated to be a reliable method to detect OSCC and potentially malignant oral lesions (Park et al., 2009; Wiklund et al., 2011; Zahran et al., 2015; Greither et al., 2017; Maheswari et al., 2018). Salivary miR-139-5p has also demonstrated to be a valuable biomarker for response to therapy and evaluation of OSCC recurrence; it was downregulated in saliva samples from OSCC patients compared to the saliva of healthy subjects, and its expression increased in OSCC patient saliva after surgery (Duz et al., 2016).

**TABLE 3:** Diagnostic and follow-up application of miRNAs in OSCC, based on population study.

**Authors (year)**	**Title**	**Cases**	**Controls**	**Type of alteration**	**Application**	**Results**
Park et al., 2009	Salivary microRNA: discovery, characterization, and clinical utility for oral cancer detection	10 OSCC	10	Expression downregulation	Diagnosis	miRNAs are present in both whole saliva and supernatant saliva. Two of evaluated miRNAs, miR-125a and miR-200a, are downregulated in the saliva of the OSCC patients compared to healthy controls, being useful in detecting OSCC.
Wiklund et al., 2011	MicroRNA alterations and associated aberrant DNA methylation patterns across multiple sample types in oral squamous cell carcinoma	15 OSCC	7	Aberrant expression and DNA hypermethylation	Diagnosis	Compared to healthy subjects, OSCC patients had deregulated miRNAs with associated DNA methylation patterns. Particularly, repression of miR-375 and methylation on miR-137, miR-200c-141, and miR-200 s/miR-205 loci were found in OSCC patients vs. healthy patients, being promising candidates to develop OSCC-specific miRNA signatures.
Liu et al., 2012	Exploiting salivary miR-31 as a clinical biomarker of oral squamous cell carcinoma	45 OSCC	24	Expression upregulation	Diagnosis and follow-up	Salivary miR-31 was significantly increased in patients with OSCC at all clinical stages, including very early stages. In addition, it was shown to be more abundant in saliva than in plasma, and after tumor surgical removal, its expression was reduced. Salivary miR-31 is a promising specific biomarker for early detection and postoperative follow-up of OSCC.
Yang et al., 2013	Progress risk assessment of oral premalignant lesions with saliva miRNA analysis	45 OSCC potentially progressing LGD leukoplakia	-	Aberrant expression	Diagnosis	A specific miRNA aberrant profile was found in saliva samples of progressing oral LGD leukoplakia; it allows monitoring of cancer precursor lesions and early detection of progression toward OSCC, before clinical evidence.
Momen-Heravi et al., 2014	Genomewide study of salivary microRNAs for detection of oral cancer	9 OSCC-bt, 8 OSCC-r	9	Aberrant expression	Diagnosis	The miRNA profiles derived from OSCC, OSCC-r, and healthy controls were distinctively different. In particular, overexpression of miRNA-27b was found in OSCC saliva samples and not in the saliva of the other two groups; it can be potentially considered a promising OSCC diagnosis salivary biomarker.
Zahran et al., 2015	Salivary microRNAs in oral cancer	100 OC (20 OSCC)	20	Aberrant expression	Diagnosis	Salivary miRNA-21, miRNA-145, and miRNA-184 were differentially expressed in OSCC and healthy saliva samples. They can be considered non-invasive, rapid diagnostic biomarkers for oral malignant transformation and early detection of OSCC, with miRNA-184 having the best diagnostic value.
Al-malkey and Abbas, 2015	Expression analysis of salivary microrna-31 in oral cancer patients	35 OC (OSCC not specified)	20	Expression upregulation	Diagnosis and follow-up	Salivary miR-31 expression increased in OC patients, with enrichment in saliva compared with plasma, allowing the proposal of miR-31 as a sensitive biomarker for early detection and postsurgical follow-up of OC.
Duz et al., 2016	Identification of miR-139-5p as a saliva biomarker for tongue squamous cell carcinoma: a pilot study	25 OSCC	25	Aberrant expression	Diagnosis	Salivary miR-139-5p was significantly reduced in TSCC patients compared to controls, and its level turned back to normal after surgery. It is a feasible and promising diagnostic marker.
Greither et al., 2017	Salivary miR-93 and miR-200a as post-radiotherapy biomarkers in head and neck squamous cell carcinoma	17 HNSCC (5 OSCC)	–	Aberrant expression	Follow-up	MiR-93 and miR-200a were significantly increased 12 months after radiotherapy, demonstrating to be valuable biomarkers for treatment monitoring post-radiation of HNSCC.

*miRNA, microRNA; LGD, low-grade dysplasia; OSCC, oral squamous cell carcinoma; OSCC-bt, oral cancer cell carcinoma before treatment; OSCC-r, oral cancer cell carcinoma in remission; OC, oral cancer; TSCC, tongue squamous cell carcinoma.*

Finally, other studies also focused on the potential of salivary miRNAs to assess the progression risk of oral premalignant lesions to OSCC, distinguishing progressing from non-progressing oral low-grade dysplasia; for example, the increased expression of miR-708, miR-10b, miR-19a, miR-30e, miR-26a, and miR-660 and decreased expression of miR-99, miR-15a, miR-197, miR-145, and miR-150 in saliva of OSCC patients represent biomarkers for neoplastic malignant transformation of oral lesions as oral low-grade dysplasia; some miRNAs, notably acting in cancer as oncosuppressors and found upregulated in patients with oral low-grade dysplasia, could represent elements of protection against the malignant transformation (Yang et al., 2013). Even though current evidence on the role of miRNAs in OSCC is encouraging, it is suggested that a combination of salivary miRNAs and proteomic data could help to have greater coverage and increased robustness of results (Yakob et al., 2014; Zahran et al., 2015).

### Other Potential Biomarkers: CTCs

Circulating tumor cells have been widely investigated in the bloodstream as other potential OSCC biomarkers. They are released from primary tumor mass, disseminating via the lymphatic and blood vessels throughout the body. Their presence in blood circulation is correlated to metastasis, recurrences, and worse prognosis (Jatana et al., 2010; Hristozova et al., 2011; Alix-Panabieŕes and Pantel, 2013; Grobe et al., 2013). They can both promote the development of metastasis and colonize the primary tumor site, supporting the primary tumor growth in a process called tumor self-seeding (Ilie et al., 2014; Zhang et al., 2016). Many studies highlighted the correlation between increased levels of CTCs and OSCC diagnosis (Nichols et al., 2012; He et al., 2013); however, it seems that the increased number of CTCs is more correlated to prognosis and locoregional relapse (Jatana et al., 2011; Buglione et al., 2012; Gröbe et al., 2014). Therefore, analysis of CTCs in blood samples of cancer patients may really support clinician decisions, helping to phenotypically and genetically characterize the primary tumor (Jatana et al., 2010; Ilie et al., 2014; Lianidou et al., 2014; Rolfo et al., 2014; Tinhofer et al., 2014; Wu et al., 2016; Zhou et al., 2016), but being more frequently found in late-stage cancers, it seems to be more informative as a tool for cancer prognosis; moreover, the investigation of drug molecular targets on the CTC surface could help to develop immunotherapies (Nonaka and Wong, 2018). All studies performed so far derived from analysis of blood samples; the investigation of CTC on saliva remains unexplored. We conceive that future studies on salivary CTCs from OSCC patients may provide relevant information about tumor prognosis and genetic alterations potentially associated with tumor recurrence, contributing to development of targeted therapies.

## Discussion

With a frequency of 2–4% of all worldwide cancer cases, OSCC is the most common epithelial neoplasia affecting the head-and-neck area (Markopoulos, 2012). Many efforts have been done so far to improve the strategies of OSCC diagnosis, prognosis, and development of therapies; however, it still is a cancer with poor prognosis (Moore et al., 2000). This primarily depends on diagnostic delay and insufficiently informative techniques to do prognosis and uncover tumor recurrence after surgery and/or therapy establishment. Given the intra-tumoral and inter-tumoral heterogeneity and the dynamic behavior with modification over time on the molecular profile, also depending on the therapeutical response, the traditional strategies of cancer screening are not sufficient for the effective management of OSCC; we urgently need novel and more effective time-saving strategies (Bellairs et al., 2017). In light of the above, the latest body of literature has focused on understanding the tumor-leading molecular basis, to develop more precise and advanced methodologies for early detection, prognosis, and establishment of successful therapies, decreasing the rate of recurrences (Woolgar, 2006).

Liquid biopsy is currently one of the most investigated techniques to easily outline the molecular identity of cancers in both early and late stages, and due to its accessibility, ease of employment, and natural feature of being in close contact with OSCC cells, saliva is considered one of the most indicative body fluids for liquid biopsy in OSCC (Kaczor-Urbanowicz et al., 2017; Gai et al., 2018; Khurshid et al., 2018).

Saliva has been proposed as a valuable means for early diagnosis, definition of cancer patient molecular profile, and development of a personalized therapy and allows monitoring of response and cancer relapse; indeed, it contains bioactive elements such as ctDNAs, miRNAs, and EVs derived from OSCC cells, where genetic and epigenetic alterations can be easily detected and give relevant information. In particular, salivary ctDNA, miRNAs, and EVs seem to be the preferred biomarkers for early detection and diagnosis; instead, CTCs, even if more widely investigated in plasma, seem to be more informative to predict prognosis (Nonaka and Wong, 2018).

The results obtained so far lead to the term “salivaomics” to indicate all the translational and clinical tools based on salivary biomarkers and considered very supportive for cancer management (Wong, 2014).

## Conclusion and Future Perspectives

There is an increasing interest in employing saliva samples as a source of biomarkers for OSCC liquid biopsy analysis. However, there are still several challenges that are yet to be overcome which are briefly indicated below and summarized in Table 4 for each of the mentioned and revised biomarkers.

**TABLE 4:** Pros and cons of salivary biomarkers in OSCC diagnosis, prognosis and follow-up.

**Salivary biomarkers**	**Pros**	**Cons**
**ctDNA**	• ctDNA holds cancer DNA genetic and epigenetic alterations	• Inter-patient (e.g., age, gender, diet, and smoking) and intra-patient variability and tumoral and temporal heterogeneity
		• Non-tumoral circulating free DNA contamination of sample
	• ctDNA can reflect the complex molecular profile deriving from tumor spatial and intratumoral heterogeneity	• High costs of isolation and detection technologies (e.g., NGS and RT-qPCR)
	• DNA alterations in ctDNA and/or relative levels are useful for cancer diagnosis, prognosis, and follow-up	• Little coverage of ctDNA alteration detection platform
		• No standardized and reproducible protocols
**EVs**	• EVs allow detection of low-expression biomarkers otherwise not detectable in saliva	• Complex and expensive isolation (i.e., ultracentrifugation, ExoQuick-TC, and an aqueous two-phase system), characterization (e.g., electron microscopy, dynamic light scattering, and western blotting), and analysis technologies (e.g., RT-qPCR)
	• EV different relative levels, morphology, and composition (e.g., lipids, proteins, DNA, and miRNAs) are useful for cancer diagnosis and prognosis	• No standardized and reproducible protocols
**miRNAs**	• The aberrant expression of miRNAs is informative for cancer diagnosis and follow-up	• Expensive detection and analysis (e.g., RT-qPCR) technologies
		• Absence of valid endogenous RT-qPCR control
		• Inter-patient variability (e.g., age and inflammation)
		• No standardized and reproducible protocols

*ctDNA, circulating tumor DNA; EV, extracellular vesicle; miRNA, microRNA; NGS, next-generation sequencing; OSCC, oral squamous cell carcinoma; RT-qPCR, quantitative reverse-transcription PCR.*

### ctDNA

Salivary ctDNA holds cancer DNA genetic and epigenetic alterations; it can reflect the complex molecular profile deriving from tumor spatial and intratumoral heterogeneity, and its alterations in sequence can primarily support diagnosis and secondly prognosis and assessment of patient response to therapy; nevertheless, several challenges still need to be overcome for ctDNA implementation in clinics (Van Ginkel et al., 2017; Payne et al., 2018). Firstly, it is necessary to improve the ctDNA isolation methodologies and sensibility of detection technologies to remove the noise due to cfDNA molecules physiologically released from non-tumoral cells and detect cancer even in the presence of an almost undetectable amount of ctDNA. In addition, we need to reduce the bias derived from inter-patient (e.g., age, gender, diet, and smoking) and intra-patient variability and tumoral and temporal heterogeneity, for a higher specificity of analysis, and it is necessary to improve the genetic coverage of ctDNA detection platforms to detect multiple molecular markers (Ignatiadis et al., 2015).

### EVs

Extracellular vesicles allow detection of low-expression biomarkers, and the relative levels, morphology, and composition (e.g., lipids, proteins, and nucleic acids) were informative for oral cancer diagnosis and prognosis. Nevertheless, the use of EVs in OSCC management still has limitations related to the complex and expensive isolation, characterization, and analysis technologies. Among all methods to isolate salivary EVs, ultracentrifugation and ExoQuick-TC (a chemical-based agent kit used to precipitate EVs) represent the most currently physical-based used methods. Ultracentrifugation is a widely used isolation methodology able to give a minimally contaminated EV pellet; however, it is a complex and prolonged process that needs specialized and expensive equipment as well as large samples. In contrast, ExoQuick-TC can isolate and purify EVs from small samples, even if with more impurities compared to ultracentrifugation (Théry et al., 2006; Zlotogorski-Hurvitz et al., 2015; Boriachek et al., 2018). Another technique to isolate salivary EVs, named the aqueous two-phase system, has been recently developed by Kim et al. (2017) and seems to be more efficient in terms of yield and purity compared to ultracentrifugation and ExoQuick-TC. With regard to characterization, the following techniques are worth mentioning: electron microscopy; an ultra-sensitive technique of low-force microscopy to investigate shape, structure, chemistry, and mechanics of vesicles; dynamic light scattering to perform a size distribution analysis; and western blotting analysis to evaluate EV-specific markers (Théry et al., 2006; Byun et al., 2015). However, despite their widespread use in daily laboratory activity, the above-described techniques are still too expensive and time-consuming to employ in clinical routine. The use of size and surface markers for selection is urgently necessary to increase yield and quality of isolated vesicles (Witwer, 2015; Barger et al., 2016); besides, the development of new and innovative strategies is essential to more easily characterize EVs. Last, but not least, the validation and standardization of protocols are essential to safely move from the bench to the bedside; develop an easy, cost-effective, and robust clinical strategy; and obtain reproducible data (Witwer et al., 2013; Zahran et al., 2015; Han et al., 2018).

### MiRNAs

The aberrant expression of miRNAs is very supportive of OSCC diagnosis, but as previously described for other salivary biomarkers, we still need to face many challenges. The protocols used to analyze and quantify isolated miRNAs need to be normalized and standardized. In the case of RT-qPCR analysis, global mean expression and exogenous (spiked-in) miRNAs and endogenous controls are currently used to normalize RNAs (Mestdagh et al., 2009; Yoshizawa and Wong, 2013). Current research identified miR-191 and miR-16 as unique suitable endogenous controls; however, they need to be validated in larger cohorts of patients (Momen-Heravi et al., 2014). Another variable that influences miRNA investigation in OSCC is the inter-patient variability (e.g., age and inflammation). Age represents a continuous variable affecting the investigations; many studies demonstrate that miRNAs are involved in the regulation of aging processes and are often upregulated in older individuals. This may be limiting and decrease the robustness, sensitivity, and specificity of miRNA investigation in OSCC patients (Wan et al., 2017). Inflammation is a typical condition of cancer development and influences the expression of miRNAs; possible fluctuations of miRNA expression may not be directly linked to cancer features, but to body inflammatory response to cancer presence, perturbing miRNA expression and decreasing reproducibility of data (Kulkarni et al., 2017). Standardization of protocols should be defined to store and handle isolated miRNAs, making them available for clinical routine (Witwer et al., 2013).

## Conclusion

In line with the current status and perspectives of liquid biopsy and saliva analysis, saliva has promising implications in OSCC management; it is conceivable that future studies on employment of ctDNAs, EVs, miRNAs, and CTCs, as salivary biomarkers in OSCC clinical routine, will certainly help to establish consistent strategies for early diagnosis of cancer lesions, facilitate advance prevention, and support the development of targeted therapies; this will improve treatment outcomes of OSCC patients, also reducing chemotherapy/radiotherapy side effects.

## Author Contributions

MC, VP, and RM conceptualized the manuscript, reviewed the literature, and drafted the manuscript. OD, GG, and GC reviewed the manuscript, critically edited, and intellectually contributed.

## Conflict of Interest

The authors declare that the research was conducted in the absence of any commercial or financial relationships that could be construed as a potential conflict of interest. The handling Editor declared a past co-authorship with one of the authors, GC.

## References

[B1] Alix-Panabieŕes C. Pantel K. (2013). Circulating tumor cells: liquid biopsy of cancer. *Clin. Chem.* 59 110–118. 10.1373/clinchem.2012.194258 23014601

[B2] Al-malkey M. K. Abbas A. A. H. (2015). Original research article expression analysis of salivary microrna-31 in oral cancer patients. *Int. J. Curr. Microbiol. Appl. Sci.* 4 375–382.

[B3] Aro K. Wei F. Wong D. T. Tu M. (2017). Saliva liquid biopsy for point-of-care applications. *Front. Public Heal.* 5:77. 10.3389/fpubh.2017.00077 28443278 PMC5387045

[B4] Barger J. F. Rahman M. A. Jackson D. Acunzo M. Nana-Sinkam S. P. (2016). Extracellular miRNAs as biomarkers in cancer. *Food Chem. Toxicol.* 98(Pt A), 66–72. 10.1016/j.fct.2016.06.010 27311798 PMC5086286

[B5] Bartel D. P. (2004). MicroRNAs: genomics, biogenesis, mechanism, and function. *Cell* 116 281–297. 10.1016/S0092-8674(04)00045-5 14744438

[B6] Bellairs J. A. Hasina R. Agrawal N. (2017). Tumor DNA: an emerging biomarker in head and neck cancer. *Cancer Metastasis Rev.* 36 515–523. 10.1007/s10555-017-9685-x 28801876 PMC5839661

[B7] Boriachek K. Islam M. N. Möller A. Salomon C. Nguyen N. T. Hossain M. S. A., et al. (2018). Biological functions and current advances in isolation and detection strategies for exosome nanovesicles. *Small* 14. 10.1002/smll.201702153 29282861

[B8] Buglione M. Grisanti S. Almici C. Mangoni M. Polli C. Consoli F. (2012). Circulating tumour cells in locally advanced head and neck cancer: preliminary report about their possible role in predicting response to non-surgical treatment and survival. *Eur. J. Cancer* 48 3019–3026. 10.1016/j.ejca.2012.05.007 22682019

[B9] Byun J. S. Hong S. H. Choi J. K. Jung J. K. Lee H. J. (2015). Diagnostic profiling of salivary exosomal microRNAs in oral lichen planus patients. *Oral Dis.* 21 987–993. 10.1111/odi.12374 26389700

[B10] Carvalho A. L. Henrique R. Jeronimo C. Nayak C. S. Reddy A. N. Hoque M. O. (2011). Detection of promoter hypermethylation in salivary rinses as a biomarker for head and neck squamous cell carcinoma surveillance. *Clin. Cancer Res.* 17 4782–4789. 10.1158/1078-0432.CCR-11-0324 21628494 PMC3215270

[B11] Chan K. C. A. Zhang J. Hui A. B. Y. Wong N. Lau T. K. Leung T. N. (2004). Size distributions of maternal and fetal DNA in maternal plasma. *Clin. Chem.* 50 88–92. 10.1373/clinchem.2003.024893 14709639

[B12] Chang Y. Tolani B. Nie X. Zhi X. Hu M. He B. (2017). Review of the clinical applications and technological advances of circulating tumor DNA in cancer monitoring. *Ther. Clin. Risk Manag.* 13 1363–1374. 10.2147/TCRM.S141991 29066904 PMC5644666

[B13] Cheng F. Su L. Qian C. (2016). Circulating tumor DNA: a promising biomarker in the liquid biopsy of cancer. *Oncotarget* 7 48832–48841. 10.18632/oncotarget.9453 27223063 PMC5217053

[B14] Chiappin S. Antonelli G. Gatti R. De Palo E. F. (2007). Saliva specimen: a new laboratory tool for diagnostic and basic investigation. *Clin. Chim. Acta* 383 30–40. 10.1016/j.cca.2007.04.011 17512510

[B15] Corrado C. Raimondo S. Chiesi A. Ciccia F. De Leo G. Alessandro R. (2013). Exosomes as intercellular signaling organelles involved in health and disease: basic science and clinical applications. *Int. J. Mol. Sci.* 14 5338–5366. 10.3390/ijms14035338 23466882 PMC3634447

[B16] Demokan S. Chang X. Chuang A. Mydlarz W. K. Kaur J. Huang P. (2010). KIF1A and EDNRB are differentially methylated in primary HNSCC and salivary rinses. *Int. J. Cancer* 127 2351–2359. 10.1002/ijc.25248 20162572 PMC2946472

[B17] Deutsch O. Fleissig Y. Zaks B. Krief G. Aframian D. J. Palmon A. (2008). An approach to remove alpha amylase for proteomic analysis of low abundance biomarkers in human saliva. *Electrophoresis* 29 4150–4157. 10.1002/elps.200800207 18937257

[B18] Di Meo A. Bartlett J. Cheng Y. Pasic M. D. Yousef G. M. (2017). Liquid biopsy: a step forward towards precision medicine in urologic malignancies. *Mol. Cancer* 16:80. 10.1186/s12943-017-0644-5 28410618 PMC5391592

[B19] Diehl F. Schmidt K. Choti M. A. Romans K. Goodman S. Li M. (2008). Circulating mutant DNA to assess tumor dynamics. *Nat. Med.* 14 985–990. 10.1038/nm.1789 18670422 PMC2820391

[B20] Dionne K. R. Warnakulasuriya S. Zain R. B. Cheong S. C. (2015). Potentially malignant disorders of the oral cavity: current practice and future directions in the clinic and laboratory. *Int. J. Cancer* 136 503–515. 10.1002/ijc.28754 24482244

[B21] Duz M. B. Karatas O. F. Guzel E. Turgut N. F. Yilmaz M. Creighton C. J. (2016). Identification of miR-139-5p as a saliva biomarker for tongue squamous cell carcinoma: a pilot study. *Cell. Oncol.* 39 187–193. 10.1007/s13402-015-0259-z 26650483 PMC13001845

[B22] El Andaloussi S. Mäger I. Breakefield X. O. Wood M. J. A. (2013). Extracellular vesicles: biology and emerging therapeutic opportunities. *Nat. Rev. Drug Discov.* 12 347–357. 10.1038/nrd3978 23584393

[B23] El-Naggar A. K. Mao L. Staerkel G. Coombes M. M. Tucker S. L. Luna M. A. (2001). Genetic heterogeneity in saliva from patients with oral squamous carcinomas: implications in molecular diagnosis and screening. *J. Mol. Diagn.* 3 164–170. 10.1016/S1525-1578(10)60668-X 11687600 PMC1906964

[B24] Ferlazzo N. Currò M. Zinellu A. Caccamo D. Isola G. Ventura V. (2017). Influence of MTHFR genetic background on p16 and MGMT methylation in oral squamous cell cancer. *Int. J. Mol. Sci.* 18 E724. 10.3390/ijms18040724 28353639 PMC5412310

[B25] Francis G. Stein S. (2015). Circulating cell-free tumour DNA in the management of cancer. *Int. J. Mol. Sci.* 16 14122–14142. 10.3390/ijms160614122 26101870 PMC4490543

[B26] Fuller C. Camilon R. Nguyen S. Jennings J. Day T. Gillespie M. B. (2015). Adjunctive diagnostic techniques for oral lesions of unknown malignant potential: systematic review with meta-analysis. *Head Neck* 37 755–762. 10.1002/hed.23667 24596227

[B27] Gai C. Camussi F. Broccoletti R. Gambino A. Cabras M. Molinaro L. (2018). Salivary extracellular vesicle-associated miRNAs as potential biomarkers in oral squamous cell carcinoma. *BMC Cancer* 18:439. 10.1186/s12885-018-4364-z 29669525 PMC5907383

[B28] Greither T. Vorwerk F. Kappler M. Bache M. Taubert H. Kuhnt T. (2017). Salivary miR-93 and miR-200a as post-radiotherapy biomarkers in head and neck squamous cell carcinoma. *Oncol. Rep.* 38 1268–1275. 10.3892/or.2017.5764 28677748

[B29] Grobe A. Blessmann M. Hanken H. Friedrich R. E. Schon G. Wikner J. (2013). Prognostic relevance of circulating tumor cells in blood and disseminated tumor cells in bone marrow of patients with squamous cell carcinoma of the oral cavity. *Clin. Cancer Res.* 20 425–433. 10.1158/1078-0432.CCR-13-1101 24218516

[B30] Gröbe A. Blessmann M. Hanken H. Friedrich R. E. Schön G. Wikner J. (2014). Prognostic relevance of circulating tumor cells in blood and disseminated tumor cells in bone marrow of patients with squamous cell carcinoma of the oral cavity. *Clin. Cancer Res.* 20 425–433. 10.1158/1078-0432.CCR-13-1101 24218516

[B31] Guerrero-Preston R. Soudry E. Acero J. Orera M. Moreno-López L. Macía-Colón G. (2011). NID2 and HOXA9 promoter hypermethylation as biomarkers for prevention and early detection in oral cavity squamous cell carcinoma tissues and saliva. *Cancer Prev. Res.* 4 1061–1072. 10.1158/1940-6207.CAPR-11-0006 21558411 PMC3131432

[B32] Han Y. Jia L. Zheng Y. Li W. (2018). Salivary exosomes: emerging roles in systemic disease. *Int. J. Biol. Sci.* 14 633–643. 10.7150/ijbs.25018 29904278 PMC6001649

[B33] He S. Li P. He S. Long T. Zhang N. Fang J. (2013). Detection of circulating tumour cells with the cellsearch system in patients with advanced-stage head and neck cancer: preliminary results. *J. Laryngol. Otol.* 127 788–793. 10.1017/S0022215113001412 23835309

[B34] Hristozova T. Konschak R. Stromberger C. Fusi A. Liu Z. Weichert W. (2011). The presence of circulating tumor cells (CTCs) correlates with lymph node metastasis in nonresectable squamous cell carcinoma of the head and neck region (SCCHN). *Ann. Oncol.* 22 1878–1885. 10.1093/annonc/mdr130 21525401

[B35] Ignatiadis M. Lee M. Jeffrey S. S. (2015). Circulating tumor cells and circulating tumor DNA: challenges and opportunities on the path to clinical utility. *Clin. Cancer Res.* 21 4786–4800. 10.1158/1078-0432.CCR-14-1190 26527805

[B36] Ilie M. Hofman V. Long E. Bordone O. Selva E. Washetine K. (2014). Current challenges for detection of circulating tumor cells and cell-free circulating nucleic acids, and their characterization in non-small cell lung carcinoma patients. What is the best blood substrate for personalized medicine? *Ann. Transl. Med.* 2:107. 10.3978/j.issn.2305-5839.2014.08.11 25489581 PMC4245510

[B37] Jahr S. Hentze H. Englisch S. Hardt D. Fackelmayer F. O. Hesch R. D. (2001). DNA fragments in the blood plasma of cancer patients: quantitations and evidence for their origin from apoptotic and necrotic cells. *Cancer Res.* 61 1659–1665. 11245480

[B38] Jankowska A. K. Waszkiel D. Kowalczyk A. (2007). Saliva as a main component of oral cavity ecosystem. Part I. Secretion and function. *Wiad. Lek.* 60 148–154. 17726867

[B39] Jatana K. R. Balasubramanian P. Lang J. C. Yang L. Jatana C. A. White E. (2010). Significance of circulating tumor cells in patients with squamous cell carcinoma of the head and neck: initial results. *Arch. Otolaryngol. Head Neck Surg.* 136 1274–1279. 10.1001/archoto.2010.223 21173379 PMC3740520

[B40] Jatana K. R. Lang J. C. Chalmers J. J. (2011). Identification of circulating tumor cells: a prognostic marker in squamous cell carcinoma of the head and neck? *Futur. Oncol.* 7 481–484. 10.2217/fon.11.19 21463135 PMC4266857

[B41] Kaczor-Urbanowicz K. E. Martin Carreras-Presas C. Aro K. Tu M. Garcia-Godoy F. Wong D. T. W. (2017). Saliva diagnostics – current views and directions. *Exp. Biol. Med.* 242 459–472. 10.1177/1535370216681550 27903834 PMC5367650

[B42] Kalluri R. (2016). The biology and function of exosomes in cancer. *J. Clin. Invest.* 126 1208–1215. 10.1172/JCI81135 27035812 PMC4811149

[B43] Kastelowitz N. Yin H. (2014). Exosomes and microvesicles: identification and targeting by particle size and lipid chemical probes. *Chem. Biochem.* 15 923–928. 10.1002/cbic.201400043 24740901 PMC4098878

[B44] Kaufman E. Lamster I. B. (2002). The diagnostic applications of saliva - a review. *Crit. Rev. Oral Biol. Med.* 13 197–212. 10.1177/154411130201300209 12097361

[B45] Khurshid Z. Zafar M. S. Khan R. S. Najeeb S. Slowey P. D. Rehman I. U. (2018). Role of salivary biomarkers in oral cancer detection. *Adv. Clin. Chem.* 86 23–70. 10.1016/bs.acc.2018.05.002 30144841

[B46] Kim J. Shin H. Park J. (2017). RNA in salivary extracellular vesicles as a possible tool for systemic disease diagnosis. *J. Dent. Res.* 96 938–944. 10.1177/0022034517702100 28410004

[B47] Kohler C. Barekati Z. Radpour R. Zhong X. Y. (2011). Cell-free DNA in the circulation as a potential cancer biomarker. *Anticancer Res.* 31 2623–2628. 21778314

[B48] Krief G. Deutsch O. Gariba S. Zaks B. Aframian D. J. Palmon A. (2011). Improved visualization of low abundance oral fluid proteins after triple depletion of alpha amylase, albumin and IgG. *Oral Dis.* 17 45–52. 10.1111/j.1601-0825.2010.01700.x 20604871

[B49] Kulkarni V. Uttamani J. R. Naqvi A. R. Nares S. (2017). MicroRNAs: emerging players in oral cancers and inflammatory disorders. *Tumor Biol.* 39:1010428317698379. 10.1177/1010428317698379 28459366

[B50] Lacombe J. Brooks C. Hu C. Menashi E. Korn R. Yang F. (2017). Analysis of saliva gene expression during head and neck cancer radiotherapy: a pilot study. *Radiat. Res.* 188 75–81. 10.1667/RR14707.1 28504589 PMC5552048

[B51] Languino L. R. Singh A. Prisco M. Inman G. J. Luginbuhl A. Curry J. M. (2016). Exosome-mediated transfer from the tumor microenvironment increases TGFβ signaling in squamous cell carcinoma. *Am. J. Transl. Res.* 8 2432–2437. 27347352 PMC4891457

[B52] Larrea E. Sole C. Manterola L. Goicoechea I. Armesto M. Arestin M. (2016). New concepts in cancer biomarkers: circulating miRNAs in liquid biopsies. *Int. J. Mol. Sci.* 17:E627. 10.3390/ijms17050627 27128908 PMC4881453

[B53] Lau C. Kim Y. Chia D. Spielmann N. Eibl G. Elashoff D. (2013). Role of pancreatic cancer-derived exosomes in salivary biomarker development. *J. Biol. Chem.* 288 26888–26897. 10.1074/jbc.M113.452458 23880764 PMC3772238

[B54] Lee Y. H. Wong D. T. (2009). Saliva: an emerging biofluid for early detection of diseases. *Am. J. Dent.* 22 241–248. 10.1016/j.bbi.2008.05.010 19824562 PMC2860957

[B55] Li L. Li C. Wang S. Wang Z. Jiang J. Wang W. (2016). Exosomes derived from hypoxic oral squamous cell carcinoma cells deliver miR-21 to normoxic cells to elicit a prometastatic phenotype. *Cancer Res.* 76 1770–1780. 10.1158/0008-5472.CAN-15-1625 26992424

[B56] Lianidou E. S. Strati A. Markou A. (2014). Circulating tumor cells as promising novel biomarkers in solid cancers. *Crit. Rev. Clin. Lab. Sci.* 51 160–171. 10.3109/10408363.2014.896316 24641350

[B57] Liao P. H. Chang Y. C. Huang M. F. Tai K. W. Chou M. Y. (2000). Mutation of p53 gene codon 63 in saliva as a molecular marker for oral squamous cell carcinomas. *Oral Oncol.* 36 272–276. 10.1016/S1368-8375(00)00005-1 10793330

[B58] Liu C. J. Lin S. C. Yang C. C. Cheng H. W. Chang K. W. (2012). Exploiting salivary miR-31 as a clinical biomarker of oral squamous cell carcinoma. *Head Neck*. 34 219–224. 10.1002/hed.21713 22083872

[B59] Liu T. Chen G. Sun D. Lei M. Li Y. Zhou C. (2017). Exosomes containing miR-21 transfer the characteristic of cisplatin resistance by targeting PTEN and PDCD4 in oral squamous cell carcinoma. *Acta Biochim. Biophys. Sin.* 49 808–816. 10.1093/abbs/gmx078 28910982

[B60] Lötvall J. Hill A. F. Hochberg F. Buzás E. I. Vizio D. Di Gardiner C. (2014). Minimal experimental requirements for definition of extracellular vesicles and their functions: a position statement from the International Society for extracellular vesicles. *J. Extracell. Vesicles.* 3:26913. 10.3402/jev.v3.26913 25536934 PMC4275645

[B61] Maheswari T. U. Venugopal A. Sureshbabu N. Ramani P. (2018). Salivary micro RNA as a potential biomarker in oral potentially malignant disorders: a systematic review. *Tzu Chi Med. J.* 30 55–60. 10.4103/tcmj.tcmj-114-17 29875583 PMC5968743

[B62] Malonek D. Vered M. Yahalom R. Dekel B. Zlotogorski Hurvitz A. (2018). PO-082 Salivary extracellular vesicles from oral cancer patients: exclusive signature of the infrared spectrum. *ESMO Open* 2018:3 10.1136/esmoopen-2018-eacr25.611

[B63] Markopoulos A. K. (2012). Current aspects on oral squamous cell carcinoma. *Open Dent. J.* 6 126–130. 10.2174/1874210601206010126 22930665 PMC3428647

[B64] Mascitti M. Orsini G. Tosco V. Monterubbianesi R. Balercia A. Putignano A. (2018). An overview on current non-invasive diagnostic devices in oral oncology. *Front. Physiol.* 9:1510. 10.3389/fphys.2018.01510 30410451 PMC6209963

[B65] Mestdagh P. Van Vlierberghe P. De Weer A. Muth D. Westermann F. Speleman F. (2009). A novel and universal method for microRNA RT-qPCR data normalization. *Genom. Biol.* 10:R64. 10.1186/gb-2009-10-6-r64 19531210 PMC2718498

[B66] Momen-Heravi F. Trachtenberg A. J. Kuo W. P. Cheng Y. S. (2014). Genomewide study of salivary MicroRNAs for detection of oral cancer. *J. Dent. Res.* 93(7 Suppl.), 86S–93S. 10.1177/0022034514531018 24718111 PMC4107544

[B67] Moore S. R. Johnson N. W. Pierce A. M. Wilson D. F. (2000). The epidemiology of tongue cancer: a review of global incidence. *Oral Dis.* 6 75–84. 10.1111/j.1601-0825.2000.tb00105.x 10702783

[B68] Mouliere F. Robert B. Peyrotte E. Del Rio M. Ychou M. Molina F. (2011). High fragmentation characterizes tumour-derived circulating DNA. *PLoS One* 6:e23418. 10.1371/journal.pone.0023418 21909401 PMC3167805

[B69] Nichols A. C. Lowes L. E. Szeto C. C. T. Basmaji J. Dhaliwal S. Chapeskie C. (2012). Detection of circulating tumor cells in advanced head and neck cancer using the cellsearch system. *Head Neck* 34 1440–1444. 10.1002/hed.21941 22076949

[B70] Nieuw Amerongen A. V. Veerman E. C. I. (2002). Saliva - The defender of the oral cavity. *Oral Dis.* 8 12–22. 10.1034/j.1601-0825.2002.1o816.x 11936451

[B71] Nonaka T. Wong D. T. W. (2018). Liquid biopsy in head and neck cancer: promises and challenges. *J. Dent. Res.* 97 701–708. 10.1177/0022034518762071 29513618 PMC5960882

[B72] Ono K. Eguchi T. Sogawa C. Calderwood S. K. Futagawa J. Kasai T. (2018). HSP-enriched properties of extracellular vesicles involve survival of metastatic oral cancer cells. *J. Cell Biochem.* 119 7350–7362. 10.1002/jcb.27039 29768689

[B73] Panzarella V. Pizzo G. Calvino F. Compilato D. Colella G. Campisi G. (2014). Diagnostic delay in oral squamous cell carcinoma: the role of cognitive and psychological variables. *Int. J. Oral Sci.* 6 39–45. 10.1038/ijos.2013.88 24287962 PMC3967306

[B74] Park N. J. Zhou H. Elashoff D. Henson B. S. Kastratovic D. A. Abemayor E. (2009). Salivary microRNA: discovery, characterization, and clinical utility for oral cancer detection. *Clin. Cancer Res.* 15 5473–5477. 10.1158/1078-0432.CCR-09-0736 19706812 PMC2752355

[B75] Pattani K. M. Zhang Z. Demokan S. Glazer C. Loyo M. Goodman S. (2010). Endothelin receptor type B gene promoter hypermethylation in salivary rinses is independently associated with risk of oral cavity cancer and premalignancy. *Cancer Prev. Res.* 3 1093–1103. 10.1158/1940-6207.CAPR-10-0115 20798208 PMC2945229

[B76] Payne K. Spruce R. Beggs A. Sharma N. Kong A. Martin T. (2018). Circulating tumor DNA as a biomarker and liquid biopsy in head and neck squamous cell carcinoma. *Head Neck*. 40 1598–1604. 10.1002/hed.25140 29542214

[B77] Peng M. Chen C. Hulbert A. Brock M. V. Yu F. (2017). Non-blood circulating tumor DNA detection in cancer. *Oncotarget* 8 69162–69173. 10.18632/oncotarget.19942 28978187 PMC5620327

[B78] Perron M. P. Provost P. (2008). Protein interactions and complexes in human microRNA biogenesis and function. *Front. Biosci.* 13 2537–2547. 10.1016/j.clinph.2011.06.006.A 17981733 PMC2901379

[B79] Pfaffe T. Cooper-White J. Beyerlein P. Kostner K. Punyadeera C. (2011). Diagnostic potential of saliva: current state and future applications. *Clin. Chem.* 57 675–687. 10.1373/clinchem.2010.153767 21383043

[B80] Pu D. Liang H. Wei F. Akin D. Feng Z. Yan Q. X. (2016). Evaluation of a novel saliva-based epidermal growth factor receptor mutation detection for lung cancer: a pilot study. *Thorac. Cancer* 7 428–436. 10.1111/1759-7714.12350 27385985 PMC4930962

[B81] Puttipanyalears C. Subbalekha K. Mutirangura A. Kitkumthorn N. (2014). Alu hypomethylation in smoke-exposed epithelia and oral squamous carcinoma. *Asian Pac. J. Cancer Prev.* 14 5495–5501. 10.7314/apjcp.2013.14.9.5495 24175848

[B82] Qin Z. Ljubimov V. A. Zhou C. Tong Y. Liang J. (2016). Cell-free circulating tumor DNA in cancer. *Chin. J. Cancer* 35 36. 10.1186/s40880-016-0092-4 27056366 PMC4823888

[B83] Ramadoss R. Krishnan R. Sukhija H. Balachander N. Raghavendhar K. Sen S. (2015). C-deletion in exon 4 codon 63 of p53 gene as a molecular marker for oral squamous cell carcinoma: a preliminary study. *Contemp. Clin. Dent.* 6(Suppl. 1), S227–S234. 10.4103/0976-237x.166840 26604578 PMC4632227

[B84] Rettori M. M. De carvalho A. C. Bomfim longo A. L. De oliveira C. Z. Kowalski L. P. Carvalho A. L. (2013). Prognostic significance of TIMP3 hypermethylation in post-treatment salivary rinse from head and neck squamous cell carcinoma patients. *Carcinogenesis* 34 20–27. 10.1093/carcin/bgs311 23042095

[B85] Rivera C. (2015). Essentials of oral cancer. *Int. J. Clin. Exp. Pathol.* 8 11884–11894. 10.1111/anae.12918 26617944 PMC4637760

[B86] Rolfo C. Castiglia M. Hong D. Alessandro R. Mertens I. Baggerman G. (2014). Liquid biopsies in lung cancer: the new ambrosia of researchers. *Biochim. Biophys. Acta Rev. Cancer* 1846 539–546. 10.1016/j.bbcan.2014.10.001 25444714

[B87] Ruivo C. F. Adem B. Silva M. Melo S. A. (2017). The biology of cancer exosomes: Insights and new perspectives. *Cancer Res.* 77 6480–6488. 10.1158/0008-5472.CAN-17-0994 29162616

[B88] Sethi S. Benninger M. S. Lu M. Havard S. Worsham M. J. (2009). Noninvasive molecular detection of head and neck squamous cell carcinoma: an exploratory analysis. *Diagn. Mol. Pathol.* 18 81–87. 10.1097/PDM.0b013e3181804b82 19430297 PMC2693294

[B89] Shah J. P. Gil Z. (2009). Current concepts in management of oral cancer - Surgery. *Oral Oncol.* 45 394–401. 10.1016/j.oraloncology.2008.05.017 18674952 PMC4130348

[B90] Shukla D. Kale A. D. Hallikerimath S. Yerramalla V. Subbiah V. (2013). Can quantifying free-circulating DNA be a diagnostic and prognostic marker in oral epithelial dysplasia and oral squamous cell carcinoma? *J. Oral Maxillofac. Surg.* 71 414–418. 10.1016/j.joms.2012.04.03 22749518

[B91] Siravegna G. Marsoni S. Siena S. Bardelli A. (2017). Integrating liquid biopsies into the management of cancer. *Nat. Rev. Clin. Oncol.* 14 531–548. 10.1038/nrclinonc.2017.14 28252003

[B92] Stadler Z. K. Thom P. Robson M. E. Weitzel J. N. Kauff N. D. Hurley K. E. (2010). Genome-wide association studies of cancer. *J. Clin. Oncol.* 28 4255–4267. 10.1200/JCO.2009.25.7816 20585100 PMC2953976

[B93] Sun W. Zaboli D. Wang H. Liu Y. Arnaoutakis D. Khan T. (2012). Detection of TIMP3 promoter hypermethylation in salivary rinse as an independent predictor of local recurrence-free survival in head and neck cancer. *Clin. Cancer Res.* 18 1082–1091. 10.1158/1078-0432.CCR-11-2392 22228635 PMC3288549

[B94] Théry C. Amigorena S. Raposo G. Clayton A. (2006). Isolation and characterization of exosomes from cell culture supernatants and biological fluids. *Curr. Protoc. Cell Biol.* 30 3.22.1–3.22.29. 10.1002/0471143030.cb0322s30 18228490

[B95] Tinhofer I. Konschak R. Stromberger C. Raguse J.-D. Dreyer J. H. Jöhrens K. (2014). Detection of circulating tumor cells for prediction of recurrence after adjuvant chemoradiation in locally advanced squamous cell carcinoma of the head and neck. *Ann. Oncol.* 25 2042–2047. 10.1093/annonc/mdu271 25057171

[B96] Valadi H. Ekström K. Bossios A. Sjöstrand M. Lee J. J. Lötvall J. O. (2007). Exosome-mediated transfer of mRNAs and microRNAs is a novel mechanism of genetic exchange between cells. *Nat. Cell Biol.* 9 654–659. 10.1038/ncb1596 17486113

[B97] Van Ginkel J. H. Huibers M. M. H. Noorlag R. De Bree R. Van Es R. J. J. Willems S. M. (2017). Liquid biopsy: a future tool for posttreatment surveillance in head and neck cancer? *Pathobiology*. 84 115–120. 10.1159/000452861 27974721

[B98] Viet C. T. Jordan R. C. Schmidt B. L. (2007). DNA promoter hypermethylation in saliva for the early diagnosis of oral cancer. *J. Calif. Dent. Assoc.* 35 844–849. 18240747

[B99] Viet C. T. Schmidt B. L. (2008). Methylation array analysis of preoperative and postoperative saliva DNA in oral cancer patients. *Cancer Epidemiol. Biomark. Prev.* 17 3603–3611. 10.1158/1055-9965.EPI-08-0507 19064577

[B100] Volik S. Alcaide M. Morin R. D. Collins C. Centre V. P. Centre G. S. (2016). Cell-free DNA clinical significance and utility in cancer shaped by emerging technologies. *Mol. Cancer Res.* 14 898–908. 10.1158/1541-7786.MCR-16-0044 27422709

[B101] Wan Y. Punyadeera C. Perry C. Kenny L. Vagenas D. Salazar C. (2017). Salivary miRNA panel to detect HPV-positive and HPV-negative head and neck cancer patients. *Oncotarget* 8 99990–100001. 10.18632/oncotarget.21725 29245955 PMC5725146

[B102] Wang B. G. Huang H. Y. Chen Y. C. Bristow R. E. Kassauei K. Cheng C. C. (2003). Increased plasma DNA integrity in cancer patients. *Cancer Res.* 63 3966–3968. 12873992

[B103] Wang J. Chang S. Li G. Sun Y. (2017). Application of liquid biopsy in precision medicine: opportunities and challenges. *Front. Med.* 11 522–527. 10.1007/s11684-017-0526-7 28744793

[B104] Wang X. Kaczor-Urbanowicz K. E. Wong D. T. W. (2017). Salivary biomarkers in cancer detection. *Med. Oncol.* 34:7. 10.1007/s12032-016-0863-4 27943101 PMC5534214

[B105] Wang Y. Springer S. Mulvey C. L. Silliman N. Schaefer J. Sausen M. (2015). Detection of somatic mutations and HPV in the saliva and plasma of patients with head and neck squamous cell carcinomas. *Sci. Transl. Med.* 7:293ra104. 10.1126/scitranslmed.aaa8507 26109104 PMC4587492

[B106] Wiklund E. D. Gao S. Hulf T. Sibbritt T. Nair S. Costea D. E. (2011). MicroRNA alterations and associated aberrant DNA methylation patterns across multiple sample types in oral squamous cell carcinoma. *PLoS One* 6:e27840. 10.1371/journal.pone.0027840 22132151 PMC3222641

[B107] Winck F. V. Ribeiro A. C. P. Domingues R. R. Ling L. Y. Riaño-Pachón D. M. Rivera C. (2015). Insights into immune responses in oral cancer through proteomic analysis of saliva and salivary extracellular vesicles. *Sci. Rep.* 5:16305. 10.1038/srep16305 26538482 PMC4633731

[B108] Witwer K. W. (2015). Circulating MicroRNA biomarker studies: pitfalls and potential solutions. *Clin. Chem.* 61 56–63. 10.1373/clinchem.2014.221341 25391989

[B109] Witwer K. W. Buzás E. I. Bemis L. T. Bora A. Lässer C. Lötvall J. (2013). Standardization of sample collection, isolation and analysis methods in extracellular vesicle research. *J. Extracell. Vesicles* 2:20360. 10.3402/jev.v2i0.20360 24009894 PMC3760646

[B110] Wong D. T. (2006). Salivary diagnostics powered by nanotechnologies, proteomics and genomics. *J. Am. Dent. Assoc.* 137 313–321. 10.14219/jada.archive.2006.0180 16570464

[B111] Wong D. T. W. (2014). Salivaomics. *J. Am. Dent. Assoc.* 143(10 Suppl.), 19S–24S. 10.14219/jada.archive.2012.0339 23034834

[B112] Wong D. T. W. (2015). Salivary extracellular noncoding RNA: emerging biomarkers for molecular diagnostics. *Clin. Ther.* 37 540–551. 10.1016/j.clinthera.2015.02.017 25795433 PMC4395471

[B113] Woolgar J. A. (2006). Histopathological prognosticators in oral and oropharyngeal squamous cell carcinoma. *Oral Oncol.* 42 229–239. 10.1016/j.oraloncology.2005.05.008 16150633

[B114] Wu X. Mastronicola R. Tu Q. Faure G. C. De Carvalho Bittencourt M. Dolivet G. (2016). A rare case of extremely high counts of circulating tumor cells detected in a patient with an oral squamous cell carcinoma. *BMC Cancer* 16:552. 10.1186/s12885-016-2591-8 27465596 PMC4964083

[B115] Xu L. Li J. H. Ye J. M. Duan X. N. Cheng Y. J. Xin L. (2017). A retrospective survival analysis of anatomic and prognostic stage group based on the American Joint committee on cancer 8thedition cancer staging manual in luminal B human epidermal growth factor receptor 2-negative breast cancer. *Chin. Med. J.* 130 1945–1952. 10.4103/0366-6999.211896 28776547 PMC5555129

[B116] Yakob M. Abemayor E. Wong D. T. W. Fuentes L. Wang M. B. (2014). Salivary biomarkers for detection of oral squamous cell carcinoma: current state and recent advances. *Curr. Oral Heal. Rep*. 1 133–141. 10.1007/s40496-014-0014-y 24883261 PMC4037864

[B117] Yáñez-Mó M. Siljander P. R. M. Andreu Z. Zavec A. B. Borràs F. E. Buzas E. I. (2015). Biological properties of extracellular vesicles and their physiological functions. *J. Extracell. Vesicles* 4:27066. 10.3402/jev.v4.27066 25979354 PMC4433489

[B118] Yang Y. Li Y. X. Yang X. Jiang L. Zhou Z. (2013). Progress risk assessment of oral premalignant lesions with saliva miRNA analysis. *BMC Cancer* 13:129. 10.1186/1471-2407-13-129 23510112 PMC3637283

[B119] Yoshizawa J. M. Wong D. T. W. (2013). Salivary micrornas and oral cancer detection. *Methods Mol. Biol.* 936 313–324. 10.1007/978-1-62703-83-0-24 23007518 PMC3630337

[B120] Zahran F. Ghalwash D. Shaker O. Al-Johani K. Scully C. (2015). Salivary microRNAs in oral cancer. *Oral Dis.* 936 313–324. 10.1111/odi.12340 25784212

[B121] Zhang Y. Ma Q. Liu T. Guan G. Zhang K. Chen J. (2016). Interleukin-6 suppression reduces tumour self-seeding by circulating tumour cells in a human osteosarcoma nude mouse model. *Oncotarget* 7 446–458. 10.18632/oncotarget.6371 26623559 PMC4808010

[B122] Zhou J. Huang A. Yang X. R. (2016). Liquid biopsy and its potential for management of hepatocellular carcinoma. *J. Gastrointest. Cancer* 47 157–167. 10.1007/s12029-016-9801-0 26969471

[B123] Zlotogorski-Hurvitz A. Dayan D. Chaushu G. Korvala J. Salo T. Sormunen R. (2015). Human saliva-derived exosomes: comparing methods of isolation. *J. Histochem. Cytochem.* 63 181–189. 10.1369/0022155414564219 25473095 PMC4340734

[B124] Zlotogorski-Hurvitz A. Dayan D. Chaushu G. Salo T. Vered M. (2016). Morphological and molecular features of oral fluid-derived exosomes: oral cancer patients versus healthy individuals. *J. Cancer Res. Clin. Oncol.* 142 101–110. 10.1007/s00432-015-2005-3 26115960 PMC11819233

